# Efficient MIR crosstalk reduction based on silicon-on-calcium fluoride platform with Ge/Si strip arrays

**DOI:** 10.1038/s41598-023-34116-9

**Published:** 2023-05-04

**Authors:** Nayira M. Elgammal, B. M. Younis, Mahmoud A. Gaafar, M. M. Elkholy, Mohamed Farhat O. Hameed, S. S. A. Obayya

**Affiliations:** 1 Physics Department, Faculty of Science, Menoufia University, Menoufia, Egypt; 2 Center for Photonics and Smart Materials, Zewail City of Science, Technology and Innovation, October Gardens, 6th of October City, Giza, 12578 Egypt; 3 Electronics and Communications Engineering Department, Misr Higher Institute for Engineering and Technology (MET), Mansoura, 35516 Egypt; 4 Physics Department, Faculty of Science, New Mansoura University, Dakahlia, Egypt; 5 Nanotechnology and Nanoelectronics Engineering Program, Zewail City of Science, Technology and Innovation, October Gardens, 6th of October City, Giza, 12578 Egypt

**Keywords:** Silicon photonics, Mid-infrared photonics

## Abstract

Reduction of the crosstalk (CT) between contiguous photonic components is still a big challenge in fabricating high packing density photonic integrated circuits (PICs). Few techniques to accomplish that goal have been offered in recent years but all in the near-IR region. In this paper, we report a design for realizing a highly efficient CT reduction in the MIR regime, for the first time to the best of our knowledge. The reported structure is based on the silicon-on-calcium-fluoride (SOCF) platform with uniform Ge/Si strip arrays. Using Ge strips shows better CT reduction and longer coupling length (L_*c*_) than the conventional Si based devices over a wide bandwidth in the MIR region. The effect of adding a different number of Ge and Si strips with different dimensions between two adjacent Si waveguides on the L_*c*_ and hence on the CT is analyzed using both full vectorial finite element method and 3D finite difference time domain method. An increase in the L_*c*_ by 4 orders of magnitude and 6.5 times are obtained using Ge and Si strips, respectively, compared to strips-free Si waveguides. Consequently, crosstalk suppression of − 35 dB and − 10 dB for the Ge and Si strips, respectively, is shown. The proposed structure is beneficial for high packing density nanophotonic devices in the MIR regime, such as switches, modulators, splitters, and wavelength division (de)multiplexers, which are important for MIR communication integrated circuits, spectrometers, and sensors.

## Introduction

Over the past few decades, with the fast development in nanophotonics technology, silicon photonics has gained a lot of interest, thanks to its compatibility with complementary metal-oxide semiconductors (CMOS) technology^1^. MIR wavelength region (ranging from 2 to 10 µm) offers a variety of practical applications. Consequently, it has become a hot research topic for science and industry. MIR spectral range, also called the “molecular fingerprint” spectrum, contains the significant rotating, vibrating, and absorbing peaks for most of the molecules with a spectral intensity that is thousands of times greater than that corresponding to the near-IR region^2^. Therefore, the MIR regime controls a variety of applications including biological and chemical sensing^3^, detection of gases^4^, medical diagnostics, thermal imaging^5^, environmental pollution monitoring^2^, healthcare, and industrial process control^6,7^. These outstanding features of the MIR regime attract researchers to design silicon photonics components/devices such as couplers^8^, waveguides^5^, photodetectors^9^, ring resonators^10^, modulators^11^, and sensors4. In MIR photonics, germanium is considered as one of the most important materials for several reasons^12^. In this context, Ge has a broad transparency range up to 16.7 µm^13^, a high free carrier density^14^, and a large refractive index (n = 4)^15^. So, when combined with low-index material such as Calcium Fluoride (CaF_2_), leads to high index contrast. In 2012, the first MIR Germanium on silicon (Ge-on-Si) waveguide has been revealed^16^, then waveguides with low loss (less than 1 dB/cm) have been introduced^17^. Also, Ge-on-CaF_2_ has been utilized as an efficient platform for optical waveguides^18^.

In Silicon/Germanium on insulator (S/GOI) platforms, light confinement in a small area could be easily achieved due to the high significant asymmetry in the refractive index of the core (e.g., Si, Ge) and its cladding or substrate (e.g., SiO_2_, air). SOI platform enables the building of several ultra-compact and high-performance photonic components employed in PICs^19^. However, the packing density of PICs is still low, which is a significant roadblock in developing large-scale, low-cost, and multilayer hybrid integrated circuits.

Recently, new approaches have been reported to improve the dense integration of PICs. In this regard, plasmonic waveguides^20^, metal-dielectric hybrid structures^21^, and metamaterial-based structures can be used to shrink the footprint of devices^22^. In the design of PICs, the effect of waveguides on each other must be considered. This is due to mode overlapping between neighboring waveguides, that results in some coupling and CT between the waveguides. However, when the optical modes are strongly confined, the overlap and the CT between waveguides are weak and insignificant. As a result, CT is considered an essential factor of the optical waveguides and device packing density. Hence, various crosstalk reduction techniques have been developed in recent years, such as nanophotonic cloaking^23^ and waveguide super-lattices^24^. The results indicate that the majority of CT reduction methods have been obtained at telecommunication wavelengths, 1.3 µm, and 1.55 µm. Also, subwavelength silicon strips and gratings have been introduced into the optical waveguide for controlling guided light in the PICs^25,26^. Consequently, compact coupled waveguide devices have emerged in recent years^27^. Khavasi et al*.*^25^, have added two subwavelength strips between two adjacent waveguides, where all-dielectric metamaterials generated a highly confined mode. Therefore, a noticeable increase in the L_*c*_ is induced in comparison to the strips-free case^25^. The L_*c*_ extends up to two orders of magnitude by adding three silicon strips between two neighboring waveguides when compared to the case of without strips. Yu et al. have achieved numerical results at the same wavelength and size of waveguides^28^. Furthermore, Yang et al. have improved the L_*c*_ by three orders of magnitude greater than that obtained in^28^ by introducing three nonuniform Si strips between the two waveguides^29^. It is worth noting that all of the aforementioned studies have worked in the NIR region, namely at λ = 1.55 µm based on the introduction of silicon strips or grating between standard SOI waveguides.

Silicon-on-calcium-fluoride (SOCF) platform is employed instead of standard SOI at the MIR wavelength region because of its high thermo optical coefficient and polarization sensitivity^30^. In addition, the absorption of SiO_2_ is quite high at this spectral regime^31^, therefore, CaF_2_ with n ≈ 1.4 (in the MIR spectrum) and a transmission window up to ≈ 9 µm is employed as a substrate. It is worth noting that CaF_2_ allows high index contrast and high transmission compared to other materials like sapphire (Al_2_O_3_) (n ≈ 1.7) and silicon nitride (Si_3_N_4_) (n ≈ 1.9) with transmission windows of up to ≈ 5.5 µm and ≈ 7 µm, respectively^7^. The wavelength region 3–5 µm contains atmospheric transmission windows, which allow silicon photonics to be used in light detection and ranging systems (LIDAR) and MIR communications^6^. In addition, the wavelength of 3.5 µm is used in sensing applications^32^ and microring resonators with low-loss propagation^33^.

In this paper, CT reduction approach is proposed in the MIR region based on SOCF platform, for the first time to the best of our knowledge. The reported structure consists of Si/Ge strips between adjacent waveguides to significantly suppress the crosstalk and increase the coupling length. Firstly, the effect of the geometrical parameters of SOCF based waveguides in strips-free case is carried out. Secondly, comprehensive studies are performed on the effect of adding Si and Ge subwavelength strips with various numbers and dimensions between SOCF based waveguides on the L_*c*_ and the CT. The full vectorial finite element method (FVFEM)^34–37^ via COMSOL Multiphysics software package (https://www.comsol.com) is utilized to perform the modal analysis. The 3D finite difference time domain (FDTD) method^38^ via Lumerical FDTD software package (https://www.lumerical.com) is used to investigate the light propagation through the proposed structure. The simulation results reveal that at λ = 3.5 µm, a very long L_*c*_ of 3.3 m with a low CT of − 34.65 dB is obtained using 2-Ge strips between two adjacent silicon waveguides. Furthermore, L_*c*_ of 5.32 mm with CT of − 9.83 dB is achieved in the case of 4-Si strips. The obtained CT (− 34.6 dB) is better than that reported in^39^ (− 22.38 dB), where two asymmetric Si-strips between two Si waveguides at λ = 1.55 µm were implemented. In addition, it is even better than that reported in^40^ (− 27.71 dB) where three Ge strips have been inserted between two Si waveguides at λ = 1.55 µm.

## Waveguide design and principle of operation

**Figure 1:**
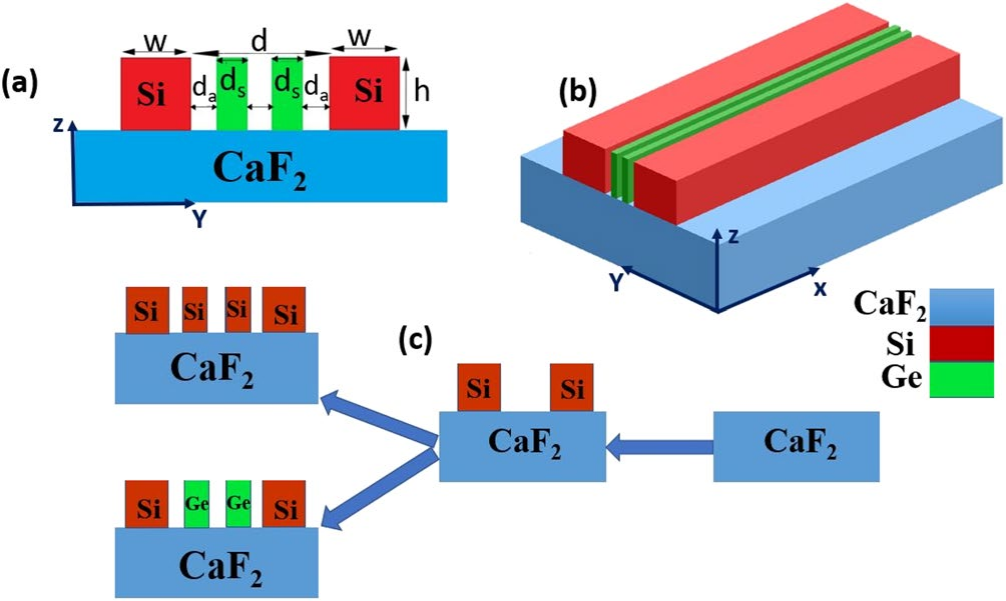
(**a**) Schematic cross-sectional view of the proposed system that comprises two symmetric Ge strip array inserted between the silicon waveguide pair, (**b**) its 3D view and (**c**) different fabrication steps of the proposed system. This image is created by Ansys Lumerical 2022 R1.3, FDTD Solver, https://www.lumerical.com (license number—675038) released to Zewail City of Science and Technology, Giza, Egypt.

The cross-sectional and 3D views of the proposed device based on SOCF platform are depicted in Figs. 1a and b, respectively. This system incorporates two subwavelength Ge or Si strips lying between two parallel silicon ridge waveguides formed on CaF_2_ substrate with air cladding. The refractive indices of Ge, Si, and CaF_2_ are obtained using Sellmeier equations reported in^41–43^. The structural parameters *w, d*_*s*_*, d*_*a*_, are the widths of the Si core, Ge or Si strips, and air slot, respectively. Moreover, *d* is the edge-to-edge distance between silicon ridge waveguides and h is the height of waveguides and strips.

The proposed system fabrication process flow is depicted in Fig. 1c. Using molecular beam epitaxy, Si layers can be grown epitaxially on a CaF_2_ substrate in ultrahigh vacuum^44^ where CaF_2_ crystal substrates are commercially on hand^45^. Using HBr/Cl_2_ chemistry, dry etching of Si can be performed^46^. It is worth mentioning that the same way can be employed to add Si and Ge array strips between the two adjacent waveguides^44^. Further, in the case of Ge strips, wafer bonding of Ge film on insulator can be employed^47^.

The CT reduction between adjacent waveguides depends on decreasing the leakage from one waveguide to its surroundings, thus increasing the L_*c*_. The L_*c*_ is defined as the distance at which the maximum optical power is transferred from one waveguide to the other. According to coupled mode theory, L_*c*_ is given by^48^:

1 $$L_{c} = \frac{\lambda }{{2\left| {n_{s} - n_{{\text{a}}} } \right|}}$$

where *n*_s_ and *n*_a_ are the real parts of the effective indices of the symmetric and anti-symmetric modes supported by the dual-core structure, respectively.

The coupling between any two adjacent waveguides can be controlled by modifying the evanescent wave penetration to the surrounding media and hence controlling the CT^23^. The evanescent wave decay constant in the second medium can be expressed as follows:

2 $$k\frac{|}{y} = \sqrt {\frac{{{\upvarepsilon }_{{\text{x}}} }}{{{\upvarepsilon }_{{\text{y}}} }}} { }\sqrt {{\upvarepsilon }_{{\text{y}}} \left( {{\text{k}}_{0} } \right)^{2} - \left( {{\text{k}}_{{\text{x}}}^{||} } \right)^{2} }$$

where, $k\frac{||}{x}$ ,$k\frac{|}{y}$ are the parallel and perpendicular components of the wave vector to the interface between the core and the surrounding medium. *ε*_*x*_, *ε*_*y*_ are the dielectric constants of the second medium parallel and perpendicular to the interface, and *k*_0_ is the wave vector in free space. From Eq. (2), the ratio of the permittivity components governs the depth of penetration of the evanescent field into the second medium. So, the decay rate of the evanescent wave for a waveguide can be managed by altering the ratio of permittivity parallel to the interface (*ε*_*x*_) to that perpendicular to it (*ε*_*y*_). As a result, penetration depth of the evanescent waves, the L_*c*_ and hence CT between two adjacent waveguides can be controlled. This can be achieved by altering the permittivity along the horizontal and the vertical directions through changing the dimensions of the strip array.

## Numerical results and discussion

**Figure 2:**
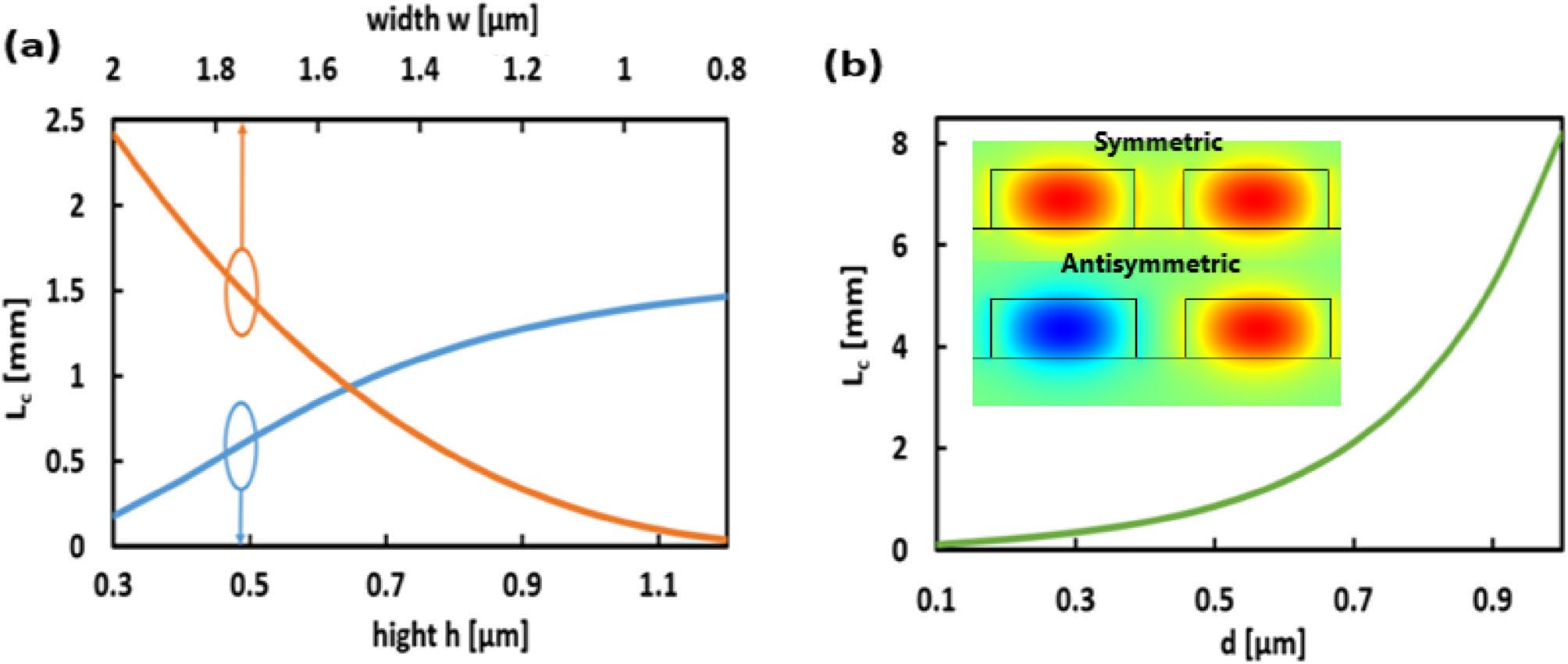
Variation of coupling length L_c_ with the change of (**a**) width *w* and height *h*, and (**b**) Variation of L_c_ with separation distance *d* of silicon waveguide without strips. Inset: electric field (E_y_) distribution (2D surface plot) of the symmetric and antisymmetric super-TE-modes for two adjacent silicon waveguides with geometrical parameters *w* = 1.5 µm, *h* = 0.6 µm, and *d* = 0.5 µm. image of Electric field distribution is created by COMSOL Multiphysics 5.3, https://www.comsol.com (license number—17074294) released to Zewail City of Science and Technology, Giza, Egypt.

Unlike SOI, which has a standard width of 500 nm and height of 220 nm, SOCF waveguides don’t have standard dimensions, especially at the MIR region. Accordingly, the geometrical parameters are studied to control the operating wavelength over the MIR regime. By changing the dimensions of the Si waveguides, the real parts of *n*_s_ and *n*_a_ modes can be controlled. Therefore, L_c_ changes according to Eq. (1). The modal analysis is performed using FVFEM. The computational domain is divided into triangular elements with a maximum element size of 3 × 10^−8^. Scattering boundary conditions are utilized in all transverse directions to truncate the simulation region. Figure 2 depicts the variation of L_*c*_ with waveguide dimensions w and h and d. It may be seen in Fig. 2a that L_c_ increases by increasing the Si waveguide’s width and height. Additionally, L_*c*_ increases with increasing d as may be seen in Fig. 2b. However, the main goal of this work is to increase the packing density of nanophotonic devices, i.e., increase the L_*c*_ with the minimum allowable waveguide’s separation. As a result, the dimensions of the SOCF-based waveguides are carefully selected to support the propagation of the strongly confined fundamental transverse electric (TE) mode as shown in the inset of Fig. 2b and obtain a minimum L_*c*_ at λ = 3.5 µm. Accordingly, the geometrical parameters of Si waveguides are taken as *w* = 1.5 µm, *h* = 0.6 µm, and *d* = 0.5 µm. The calculated L_*c*_ at these parameters is 844 µm.

Next, uniform strips of Ge or Si are added between the two adjacent waveguides to increase the L_*c*_ and reduce CT. In this investigation, the effect of the number and width of the inserted strips on the L_*c*_ and hence on the CT is studied in detail. As the type, number, and dimensions of strips change, the permittivity ratio of modes changes. By controlling this ratio, the penetration depth of the evanescent wave into the surrounding media can be managed. Consequently, the obtained CT can be controlled. Moreover, this change affects the refractive indices n_eff_ of the resultant modes. Hence, the difference in n_eff_ can be minimized to maximize L_*c*_ according to Eq. (1) and get the lowest CT value.

**Figure 3:**
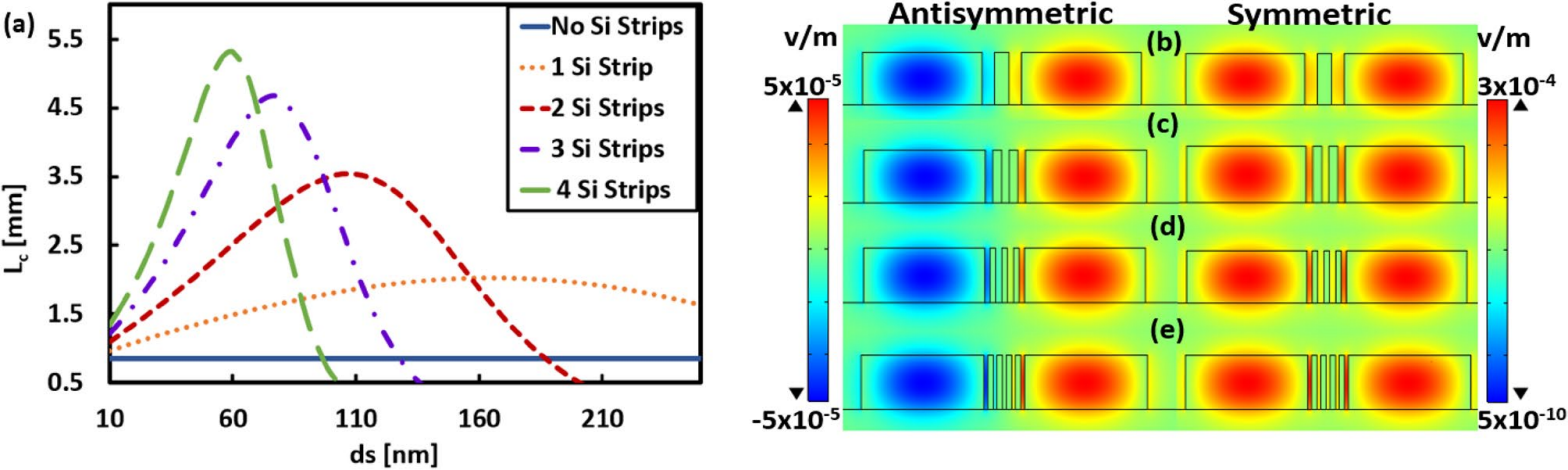
**(a)** Variation of coupling length L_*c*_ with the width *d*_*s*_ and number of Si strip and without Strip for two adjacent silicon waveguides with geometrical parameters *w* = 1.5 µm, *h* = 0.6 µm, and *d* = 0.5 µm. Electric field (E_y_) distribution (2D surface plot) of symmetric and antisymmetric TE modes for each case at effective width where L_*c*_ reaches the largest value. (**b**) 1-Si strip with width 170 nm, (**c**) 2-Si strips with width 105 nm, (**d**) 3-Si strips with width 77 nm, and (**e**) 4-Si strips with width 59 nm. The images of Electric field distribution are created by COMSOL Multiphysics 5.3, https://www.comsol.com (license number—17074294) released to Zewail City of Science and Technology, Giza, Egypt.

First, the impact of adding Si strip(s) on the L_*c*_ is studied and the results are shown in Fig. 3 and Table 1. Figure 3a shows that L_*c*_ increases by using Si-strips by a factor of 2.5 to 6.5 with respect to the case of without Si strips. Figures 3b–e depict the Electric field (E_y_) distribution (2D surface plot) of symmetric and antisymmetric TE modes at effective widths that give maximum L_*c*_ in each case. By increasing the number of Si strips array, L_*c*_ increases, and field profiles become more confined in the core region. However, by increasing the number of strips, each individual strip becomes less thick, as summarized in Table 1. It is worth noting, that reducing the thickness of strips will be a challenge in device fabrication. However, less than 50 nm gap between Si structures in Si Bragg grating waveguides have been demonstrated^49^.

**Table 1:** The effective width of different structures with Si strips and its corresponding maximum coupling length.

Two Silicon waveguide structure *λ* = 3*.*5 µm	Optimal d_*s*_ (nm)	d_*a*_ (nm)	Maximum L_*c*_ (µm)
No-Si strip	0	500	8.44 × 10^2^
1-Si strip	170	165	2.02 × 10^3^
2-Si strips	105	96.66	3.53 × 10^3^
3-Si strips	77	67.25	4.67 × 10^3^
4-Si strips	59	52.8	5.32 × 10^3^
5-Si strips	48	43.33	5.66 × 10^3^

**Figure 4:**
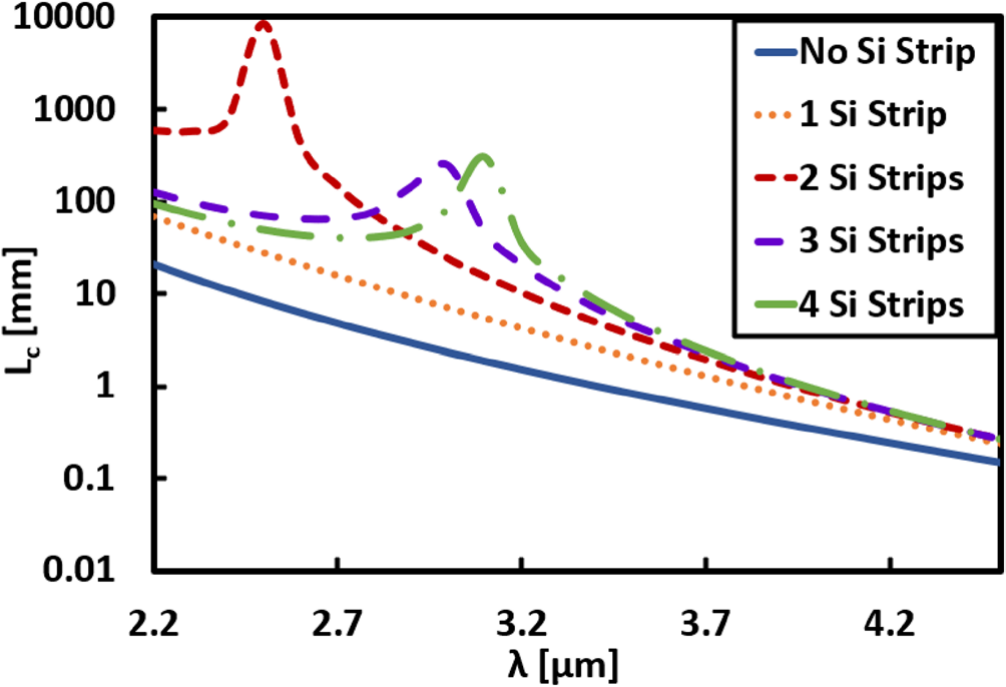
Variation of the coupling length L_*c*_ with wavelength (λ) for structures of two adjacent silicon waveguides with geometrical parameters *w* = 1.5 µm, *h* = 0.6 µm, and *d* = 0.5 µm, with and without Si strips at effective width *d*_*s*_ illustrated in Table 1.

Figure 4 illustrates the wavelength dependent L_*c*_ for different studied structures at the optimum value of d_*s*_, as summarized in Table 1. As seen from Fig. 4, L_*c*_ increases for all structures after adding thin Si strips between the two Si waveguides with respect to the case of no strips. Considering the case of no Si strips, the region between the two waveguides is uniform (air) with constant permittivity equal to 1. Thus, *ε*_*y*_ is very small, leading to a smaller decay rate $\left(k\frac{|}{y}\right)$ in the normal direction according to Eq. (2), which enhanced the coupling process between the two Si waveguides leading to small L_*c*_ throughout the whole wavelength range. However, by adding Si strips between the two waveguides, *ε*_*y*_ becomes large, then the decay rate $\left(k\frac{|}{y}\right)$ increases, and the penetration depth of the evanescent wave into the surrounding decrease. Hence the coupling between the two waveguides becomes weak, L_c_ increases. Under certain conditions, the optimum permittivity ratio is attained, L_c_ reaches a maximum value, and a peak appears in the L_c_ curve, as seen in Fig. 4. Additionally, a maximum L_c_ of 8.5 m is obtained in the case of 2-Si strips with d_s_ of 105 nm at λ = 2.5 µm. In the 3 and 4-Si strips’ structures, L_c_ has also large values of 0.25 m and 0.3 m, respectively, at λ ≃ 3 µm.

**Figure 5:**
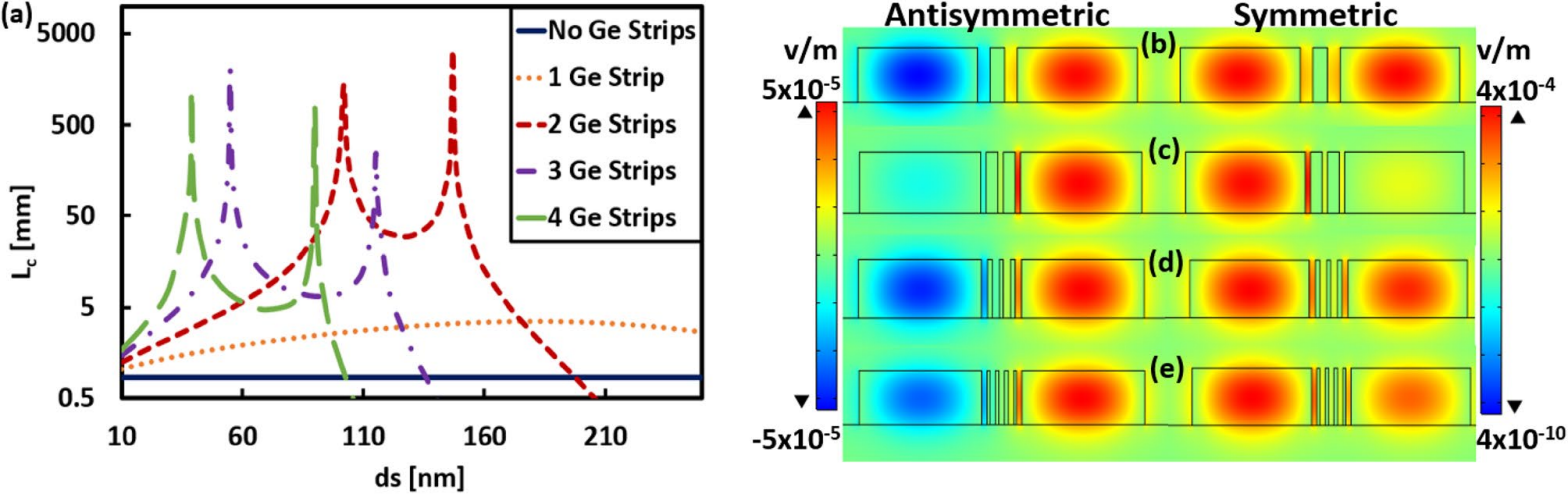
(**a**) Variation of coupling length L_*c*_ with the width *d*_*s*_ and number of Ge strip and without Strip for two adjacent silicon waveguides with geometrical parameters *w* = 1.5 µm, *h* = 0.6 µm, and *d* = 0.5 µm. Electric field (E_y_) distribution (2D surface plot) of symmetric and antisymmetric TE modes for each case at effective width where Lc reaches the largest value. (**b**) 1-Ge strip with width 180 nm, (**c**) 2-Ge strips with width 147 nm, (**d**) 3-Ge strips with width 55 nm, and (e) 4-Ge strips with width 39 nm. The images of Electric field distribution are created by COMSOL Multiphysics 5.3, https://www.comsol.com (license number—17074294) released to Zewail City of Science and Technology, Giza, Egypt.

In a similar way, variation of the L_*c*_ with the number and width of the strips is studied for Ge strips instead of Si strips with the same dimensions of Si waveguides at λ = 3.5 µm, and the obtained results are depicted in Fig. 5a. It is evident from this figure that there is a significant increase in L_*c*_ for all cases of adding Ge strip(s) between the two adjacent Si ridge waveguides relative to no used strips. Figures 5b–e depict the Electric field (E_y_) distribution (2D surface plot) for the analyzed structures with effective width that provides maximum L_*c*_. In the case of a single Ge strip, L_*c*_ becomes ≈ 4 times larger than that in the case of no strips. While for other cases (2, 3, and 4-Ge strips), the L_*c*_ increases by 4 orders of magnitude, which is considered a very high increase as illustrated in Fig. 5a and Table 2. This large increase in L_*c*_ is due to the small difference in n_eff_ between the symmetric and antisymmetric modes at the optimum width. The suggested device with Ge strips provides a much wider range of permittivity ratio. For the designs with 2, 3, and 4 Ge strips, the L_*c*_ curves have two peaks as may be seen in Fig. 5a. Because of reaching close to the optimal permittivity ratio at the effective width of Ge strips. The L_*c*_ reaches the maximum value of 3.3 m when adding 2-Ge strips between the two Si waveguides with optimum width of 147 nm. In this case, the difference in n_eff_ is very small about 1 × 10^−6^, and hence the L_*c*_ is extremely large (Eq. 1), allowing waveguides to be placed close to each other with a negligible CT. For more explanation, Table 3 shows the Electric field (E_y_) distribution (2D surface plot) and Electric field strength (1D line plot) of symmetric and antisymmetric TE modes at different widths (*d*_*s*_) of the structure of 2-Ge strips. The utilized geometrical parameters are *w* = 1.5 µm, *h* = 0.6 µm, and *d* = 0.5 µm. As shown in Table 3, when *d*_*s*_ of the 2 uniform Ge-strips changes, L_*c*_ and the field strength also change. The coupling between any two adjacent waveguides can be controlled by altering the permittivity along the horizontal and vertical directions by changing the strips' type (material itself), number, and dimensions. At optimal conditions, the coupling between two waveguides becomes weak, and the coupling length will be very large. This explains why in Fig. 5a the L_*c*_ curve corresponding to the case of 2-Ge strips has two peaks, at *d*_*s*_ = 147 nm and 102 nm. In addition, this interprets why the field strength at one core is high (i.e., E-field is well confined), and the other is weak at 102 nm and especially at 147 nm (Fig. 5c) compared to other studied widths as illustrated in Table 3. However, in the cases of non-optimal strip widths (all rows of Table 3 except the 2nd and 4th rows), the field is divided between the two cores showing a strong coupling between them. This result is revealed by the L_*c*_s depicted in the right column of Table 3. An important notice is that with Ge strips of *ds* = 147 nm, the aspect ratio of the design becomes ≈ 1:4, which is feasible via standard nanofabrication methods. However, the L_*c*_ decreases with a relative increase in the width of Ge strips (e.g., ≥ 190 nm for 1-Ge Strip) as the air slots between strips and waveguides becomes very thin, which in turn increases the evanescent wave overlapping.

**Table 2:** The effective width of different structures with Ge strips and its corresponding maximum coupling length.

Two Silicon waveguide structure λ = 3.5 µm	Optimal d_*s*_ (nm)	d_*a*_ (nm)	Maximum L_*c*_ (µm)
No-Ge strip	0	500	8.44 × 10^2^
1-Ge strip	180	160	3.52 × 10^3^
2-Ge strips	147	68.66	3.30 × 10^6^
3-Ge strips	55	83.75	2.07 × 10^6^
4-Ge strips	39	68.8	1.22 × 10^6^
5-Ge strips	30	58.33	1.32 × 10^5^

**Table 3:** Electric field (E_y_) distribution (2D surface plot) and Electric field strength (1D line plot) of symmetric and antisymmetric TE modes at different widths (ds) of the structure of 2-Ge strips between two adjacent silicon waveguides with geometrical parameters *w* = 1.5 µm, *h* = 0.6 µm, and *d* = 0.5 µm.

d_s_ (nm)	Symmetric TE mode	Antisymmetric TE mode	Color bar	Lc (μm)
70				8.38 × 10^3^
102				1.36 × 10^6^
120				3.24 × 10^4^
147				3.30 × 10^6^
160				1.29 × 10^4^

The images of Electric field distribution and field strength are created by COMSOL Multiphysics 5.3, https://www.comsol.com (license number—17074294) released to Zewail City of Science and Technology, Giza, Egypt.

**Figure 6:**
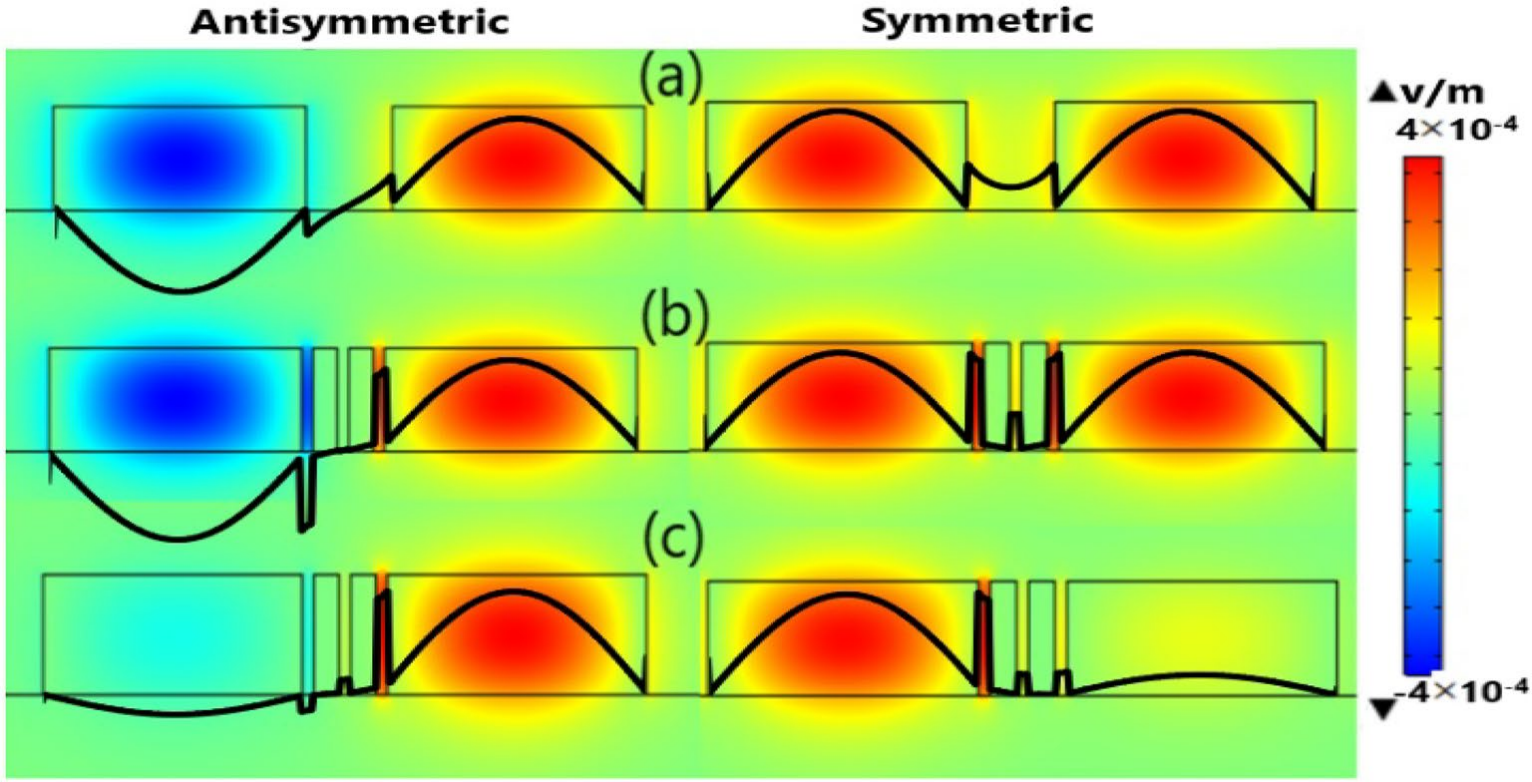
Electric field (E_y_) distribution (2D surface plot) and Electric field strength (1D line plot)) of symmetric and antisymmetric TE modes of two adjacent silicon waveguides with geometrical parameters *w* = 1.5 µm, *h* = 0.6 µm, and *d* = 0.5 µm (a) without strip (b) 2-Si strips with *d*_*s*_ = 147 nm, and (c) 2-Ge strips with *d*_*s*_ = 147 nm. The images of Electric field distribution and field strength are created by COMSOL Multiphysics 5.3, https://www.comsol.com (license number—17,074,294) released to Zewail City of Science and Technology, Giza, Egypt.

As seen from Figs. 3b–e and 5b–e, inserting Ge strips between the two Si cores instead of Si strips makes the field more confined in one core than the other. Thus, the coupling between the two Si waveguides becomes very weak by using Ge strips, and hence L_*c*_ becomes very large. To further illustrate this idea, Fig. 6. Shows the Electric field (E_y_) distribution (2D surface plot) and Electric field strength (1D line plot) of symmetric and antisymmetric TE modes for the structures of 2-Ge strips, 2-Si strips, and that of no strips. It is worth noting that this study is performed at λ = 3.5 µm where the geometrical parameters *w, h, d,* and *d*_*s*_ are taken as 1.5 µm, 0.6 µm, 0.5 µm, 147 nm, respectively. Figure 6. indicates that in the structure with 2-Ge strips the field strength at one core is high (i.e., well confined) while it is weak at the other core relative to the structure with 2-Si Strips. This means that strong coupling between the two waveguides occurs in the case of 2-Si Strips (*d*_*s*_ = 147 nm) with L_*c*_ = 2.47 × 10^3^ μm when compared to the case of 2-Ge strips (*d*_*s*_ = 147 nm) with L_*c*_ = 3.3 × 10^6^ μm in which the coupling becomes very weak. This is due to the fact that Ge has a higher refractive index (n = 4.035^41^) than Si (n = 3.4284^42^ at λ = 3.5 µm). So, in combination with CaF_2_ (n = 1.41^43^), it has a large index contrast (≈ 2.62). Furthermore, Ge has a wide range of transparency in the MIR region^12^. Therefore, adding Ge strips reduces the field penetration of optical modes from the first waveguide to the second one and hence decreases the CT level^18^.

**Figure 7:**
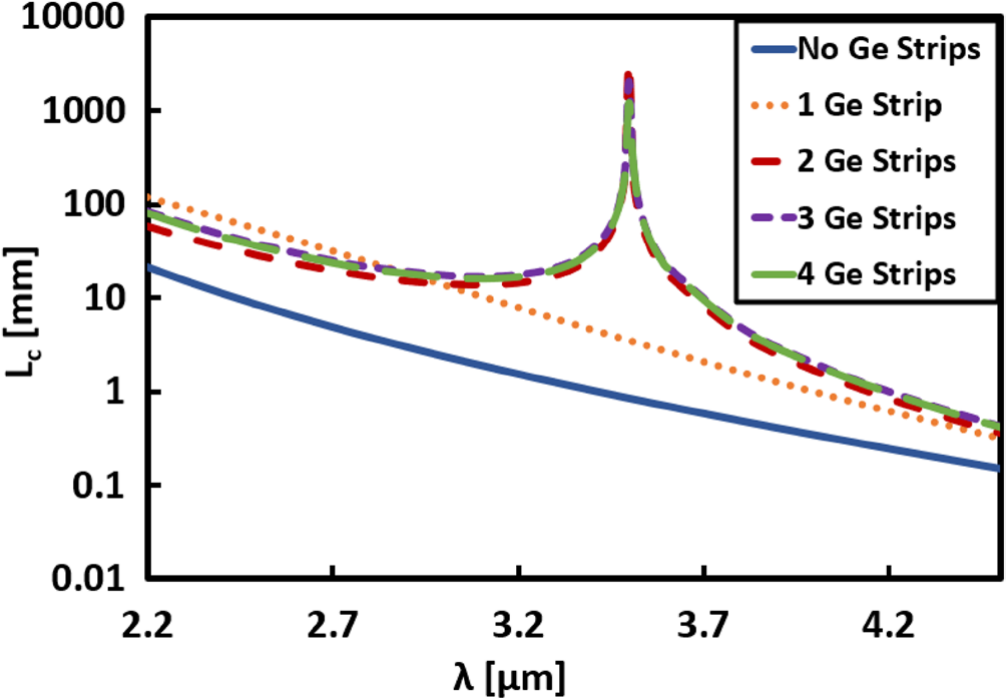
Variation of the coupling length L_*c*_ with wavelength for structures of two adjacent silicon waveguides with geometrical parameters *w* = 1.5 µm, *h* = 0.6 µm, and *d* = 0.5 µm, with and without Ge strips at effective width *d*_*s*_ illustrated in Table 2.

Coupling length analysis is then done over a wide range of wavelengths for all cases with and without Ge strips at the optimum d_*s*_ value that gives maximum L_*c*_ in each case, as shown in Fig. 7. This figure reveals that in cases of 2, 3, and 4-Ge strips, the maximum L_*c*_ is obtained exactly at λ = 3.5 µm and decreases with moving away from this wavelength. In addition, in the case of structures with zero and 1 strip, L_*c*_ is below 10 cm throughout the studied wavelength range.

It is worth mentioning that by increasing the L_*c*_, the CT decreases. CT (in dB scale) can be calculated via Eq. (3):

3 $${\text{Crosstalk}} \left( {{\text{CT}}} \right) = 10\log_{10} \left( {\frac{{{\text{P}}_{out} }}{{{\text{P}}_{in} }}} \right)$$

where P_*out*_ is the output power at the end of the second waveguide while P_*in*_ is the input power at the beginning of the first waveguide.

**Figure 8:**
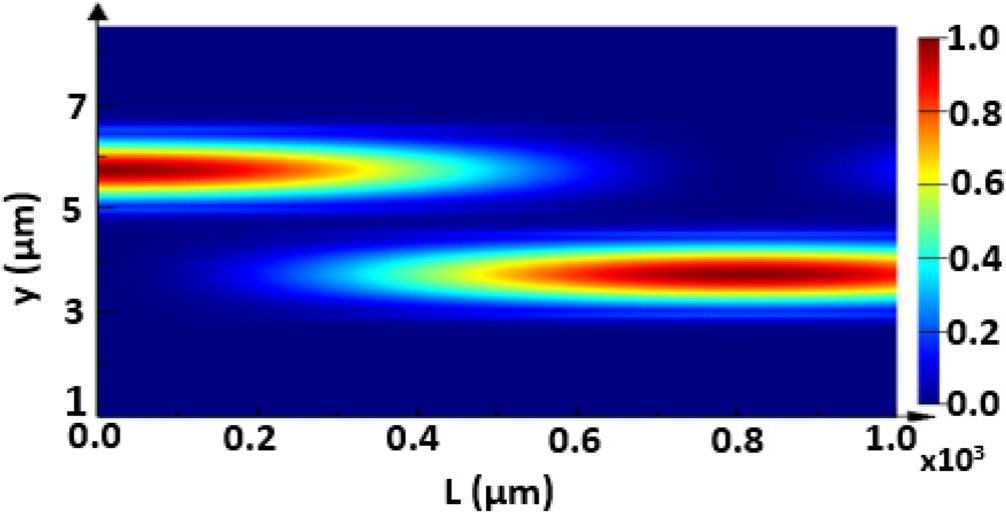
Electric field intensity distribution of the fundamental TE mode with a propagation length L = 1000 µm through the proposed structure without adding strips, where *w* = 1.5 µm, *h* = 0.6 µm, and *d* = 0.5 µm and λ = 3.5 µm. This image is created by Ansys Lumerical 2022 R1.3, FDTD Solver, https://www.lumerical.com (license number—675038) released to Zewail City of Science and Technology, Giza, Egypt.

**Figure 9:**
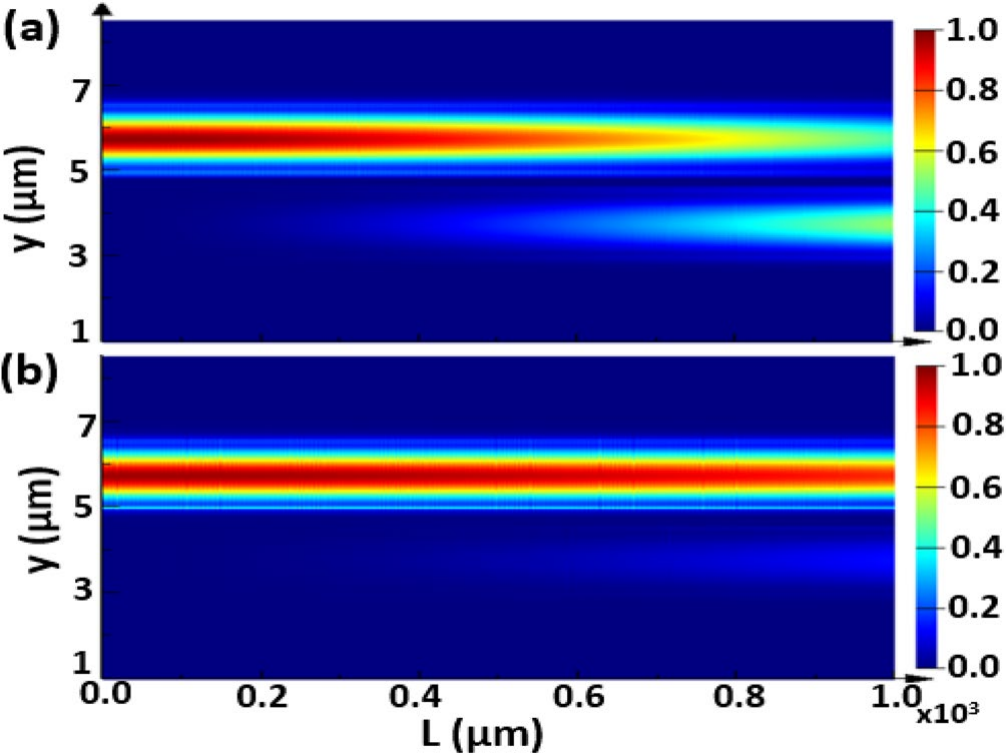
Electric field intensity distribution of the fundamental TE mode with a propagation length of L = 1000 µm through the proposed structure with adding of Si strips, where *w* = 1.5 µm, *h* = 0.6 µm, and *d* = 0.5 µm and λ = 3.5 µm. (**a**) 1-Si strip with *d*_*s*_ = 170 nm, (**b**) 4-Si strips with *d*_*s*_ = 59 nm. This image is created by Ansys Lumerical 2022 R1.3, FDTD Solver, https://www.lumerical.com (license number—675038) released to Zewail City of Science and Technology, Giza, Egypt.

**Figure 10:**
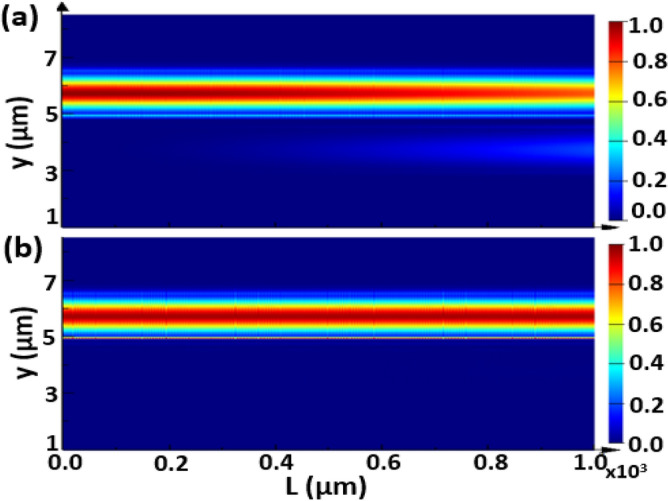
Electric field intensity distribution of the fundamental TE mode with a propagation length of L = 1000 µm through the proposed structure with adding of Ge strips, where *w* = 1.5 µm, *h* = 0.6 µm, and *d* = 0.5 µm and λ = 3.5 µm. (**a**) 1-Ge strip with *d*_*s*_ = 180 nm, (**b**) 2-Ge strips with *d*_*s*_ = 147 nm. This image is created by Ansys Lumerical 2022 R1.3, FDTD Solver, https://www.lumerical.com (license number—675038) released to Zewail City of Science and Technology, Giza, Egypt.

The propagation of the fundamental TE mode is studied at a device length of L = 1000 µm where the geometrical parameters are taken as w = 1.5 µm, h = 0.6 µm, d = 0.5 µm and λ = 3.5 µm for different cases, without adding strips and with adding Si or Ge strips as may be seen in Figs. 8, 9, and 10, respectively. The FDTD method is used to simulate the field propagation through the 3D structure via Lumerical software package. The structure is discretized into very small rectangles with auto non-uniform mesh accuracy 5 and simulation time 15,000 fs to ensure high simulation resolution. The perfectly matched layer (PML) as an absorbing boundary condition is applied to all transverse directions to truncate the simulation domain. It is evident from Fig. 8 that a complete coupling occurs using no strips between the two Si waveguides at L_*c*_ ≈ 840 µm, that agrees well with the result in Figs. 3 and Table 1. However, adding Si strips affects the coupling strength, especially with increasing the number of Si strips, as indicated in Fig. 9a and b. Figures 10 show that inserting Ge strips significantly decrease the coupling process. As illustrated in Fig. 10b, in the structure of 2-Ge strips, light can travel for more than 1000 µm with a very small CT. That is completely compatible with the results shown in Figs. 5 and Table 2 concerning the structure of 2-Ge strips which indicates that a distance L_*c*_ of 3.3 m is required for complete coupling between the two waveguides with 2-Ge strips between them.

The values of CT are calculated via Eq. (3), as indicated in Table 4, where L ≈ 840 µm and the geometrical parameters are *w* = 1.5 µm, *h* = 0.6 µm, *d* = 0.5 µm, and λ = 3.5 µm. Further, different cases, without and with adding Si and Ge strips between two Si waveguides, are considered, and the obtained CT values are summarized in Table 4. It is worth noting that L is fixed to 840 µm as a required length for complete coupling with no strips between the two adjacent waveguides. In addition, adding Ge strips is more efficient in decreasing CT than Si strips. In this context, adding 2-Ge strips decreases CT to -34.6 dB. This value of CT can be neglected, as mentioned in^50^, where photonic integration density has been evaluated by using Y-branch and optical filters. It has been reported in^50^ that if the CT between two neighboring waveguides is less than 30 dB, it can be ignored. Here, the obtained value of CT (− 34.6 dB) is better than that obtained in^39^ (− 22.38 dB), where two asymmetric Si-strips between pair of Si waveguides of width 500 nm and half-wavelength center-to-center separation (λ = 1.55 µm) has been considered. In addition, the obtained CT value in this work is better than that reported in^40^ (− 27.71 dB), where 3-Ge strips of *w* = 45 nm and *h* = 171 nm have been inserted between two Si waveguides with 500 nm width and separated by 250 nm at λ = 1.55 µm.

**Table 4:** Crosstalk of different structures with no strips and with adding Si & Ge strips between two Si waveguides.

Two Silicon waveguide structure λ = 3.5 µm, L ≈ 840 µm	Optimal d_*s*_ (nm)	d_*a*_ (nm)	Crosstalk (CT) dB
No-strip	0	500	0
1-Si strip	170	165	− 2.143
4-Si strips	59	52.8	− 9.832
1-Ge strip	180	160	− 8.1537
2-Ge strips	147	68.66	− 34.646

Based on these results, the longer L_*c*_ between Si waveguides the lower CT value. This means that the CT between waveguides is basically dependent on the coupling length. Table 5 outlines a comparison between the coupling lengths, basic platform and operating wavelength of the previously reported waveguide structures and the proposed structures. In this work, there are two reported designs based on SOCF platform instead of standard SOI. The first design is constructed by inserting Si strips between two Si waveguides in which the case of 4-Si strips gives larger L_*c*_ (5.32 mm) than those reported in^25,51^. However, in^28^ , the case of 3-Si strips in the NIR achieves a larger L_*c*_ of 332 mm. It is worth mentioning that the design based on SOCF platform with 3-Si strips of dimensions *w* = 500 nm, h = 220 nm, and *d* = 500 nm (that fit the NIR regime) offers L_c_ of 660 mm at λ = 1.55 μm. The second design depends on inserting Ge strips between two Si waveguides in which the case of 2-Ge strips gives the highest value of L_*c*_ (3.3 m) that is greater than that reported in^40^. However, in^29^, using 3 nonuniform-Si strips in the NIR has a large L_*c*_ of 312.2 m. In contrast, the method of adding uniform strips is most straightforward and simple in fabrication. Hence, the reported structure with 2-Ge strips can be utilized as a promising candidate to minimize the CT between any two nearby photonic waveguides in MIR wavelength regime.

**Table 5:** Comparison of coupling length between the suggested design and those reported in the literature.

Device	Platform	λ µm	Coupling length L_*c*_ (µm)	Remarks
Chandra et al.^40^	SOI	1.55	81.5 × 10^3^	3-Ge strips
				d = 300 nm
Yang et al.^29^	SOI	1.55	2.1 × 10^6^	(nonuniform) 3-Si strips
				d = 450 nm
			3.122 × 10^8^	d = 500 nm
Jahani et al.^51^	SOI	1.55	4 × 10^3^	5-Si strips
				d = 500 nm
Yu et al.^28^	SOI	1.55	3.32 × 10^5^	3-Si strips
				d = 500 nm
			8.5 × 10^3^	2-Si strips
Khavasi et al.^25^	SOI	1.55	3.8 × 10^3^	2-Si strips
				d = 500 nm
Proposed design	SOCF	3.5	5.32 × 10^3^	4-Si strips
				d_*s*_ = 59* nm*
				d = 500 nm
Proposed design	SOCF	3.5	3.3 × 10^6^	2-Ge strips
				d_*s*_ = 147* nm*

## Conclusion

In this paper, two designs based on the silicon-on-calcium-fluoride platform with uniform Ge/Si strip arrays are reported and analyzed to minimize the CT between two adjacent waveguides in the MIR wavelength range. The coupling length between the two fundamental modes in the two neighboring waveguides increases by increasing the decay rate of the evanescent field from the excited core. This is achieved by controlling the added strips’ material, number, and dimensions. The calculated L_*c*_ increases by factors of 2.5 to 6.5 in the case of inserting Si strips between the two adjacent Si waveguides with respect to the case of no strips achieving allow CT of − 9.83 dB. While L_*c*_ increases by 4 orders of magnitude by adding Ge strips. The maximum value of L_*c*_ = 3.3 m is obtained by adding 2-Ge strips with a very small CT of − 34.64 dB. Thus, there is almost no coupling between the two adjacent Si waveguides. To summarize, the proposed approach is beneficial for realizing a highly efficient and broadband CT reduction in the MIR regime. As a consequence, ultra-high dense PICs can be achieved in this spectral range and, therefore will pave the way for further development in different applications such as polarization splitters^52^, polarization rotators^53^, and integrated photonic switches^54^.

## Data Availability

The datasets used and/or analyzed during the current study available from the corresponding author on reasonable request.
